# Draft Genome Sequence of a *Cladosporium* Species Isolated from the Mesophotic Ascidian Didemnum maculosum

**DOI:** 10.1128/MRA.00311-20

**Published:** 2020-04-30

**Authors:** Anastasia Gioti, Romanos Siaperas, Efstratios Nikolaivits, Géraldine Le Goff, Jamal Ouazzani, Georgios Kotoulas, Evangelos Topakas

**Affiliations:** a Institute of Marine Biology, Biotechnology and Aquaculture, Hellenic Centre for Marine Research, Heraklion, Greece; b Industrial Biotechnology & Biocatalysis Group, Biotechnology Laboratory, School of Chemical Engineering, National Technical University of Athens, Athens, Greece; c Institut de Chimie des Substances Naturelles, ICSN, CNRS, Gif sur Yvette, France; Vanderbilt University

## Abstract

We report the 28-Mbp draft genome sequence of the marine fungus *Cladosporium* sp. strain TM138. The species was isolated from the marine invertebrate Didemnum maculosum. Its genome sequence will inform future investigations into the species’ enzymatic potential for bioremediation and its evolution in marine environments.

## ANNOUNCEMENT

Marine environments account for more than 95% of the total environmental biodiversity. Fungi isolated from these environments are increasingly gaining attention for their potential use in a wide spectrum of industrial applications (1) since they constitute a rich source of bioactive compounds (2, 3) and comprise an arsenal of novel enzymes that can be used in, e.g., bioremediation (1, 4). Species of *Cladosporium* exhibit broad physiological capacities, such as halotolerance (5), but relatively little is known about their genomes. We report here the draft genome sequence of a *Cladosporium* species isolated from a marine environment, obtained through shotgun sequencing. *Cladosporium* sp. strain TM138 (isolate code TM138-S3) was isolated from the yellow encrusting ascidian Didemnum maculosum, collected in March 2017 from a rock substrate in Almuñecar, Spain, at a 31-m depth (6). For isolation, a 1-cm^3^ tissue piece was ground in sterile seawater and heated at 50°C for 1 h. The suspension was serially diluted, plated onto Difco marine broth agar (MBA; BD Biosciences, NJ, USA), and incubated at 28°C for 6 weeks. Pure MBA cultures were established from a single colony. For DNA extraction, the fungal biomass was filtered from 3-day-old liquid cultures (27°C and 150 rpm shaking) in Difco marine broth, and the GenElute plant genomic DNA miniprep kit (Sigma-Aldrich, MO, USA) was used. A 450-bp insert size library was prepared with the Nextera NEBNext Ultra II FS DNA library prep kit and sequenced in paired-end mode (read length, 150 bp) by Eurofins Genomics Europe Sequencing GmbH (Constance, Germany) on a NovaSeq6000 S2 instrument.

Sequencing yielded 4,996,400 paired reads, totaling 1,498,920,000 bp. Adapter sequences were removed (at an alignment score above 7, allowing 2 mismatches), and bases with a quality score below 18 and below an average of 20 on a 5-bp window were trimmed using Trimmomatic v.0.39 (7). Reads smaller than 50 bases or with no pair (singletons) were discarded. Identification of contaminant reads was performed with Kraken2 v.0.8 (8), with a confidence score of 0.7 and using a custom database comprising UniVec and RefSeq genomes from *Bacteria*, *Archaea*, viruses, plants, and fungi (accessed December 2019). Based on this analysis, read pairs that were not classified as belonging to other taxa were used for *de novo* assembly, invoking error correction with BayesHammer as bundled with SPAdes 3.13.0 (9, 10), using k-mers 33, 55, 77, and 99, automatic computation of the coverage threshold, and an option to minimize the number of mismatches. The SPAdes assembly was polished with one round of Pilon 1.23 (11) using alignments of pairs and singletons against the genome assembly, obtained with BWA 0.7.15 (12). The genome of *Cladosporium* sp. TM138 consists of 732 scaffolds longer than 500 bp and is 28,009,899 bp long excluding gaps. The average base coverage is 48×, the *N*_50_ value is 438,704 bp, and the average G+C content is 55.69%. The genome is 95.8% complete based on BUSCO v.3.1.0 analysis (13) with the Pezizomycotina_odb90 data set, containing 3,156 ortholog genes. Gene predictions with AUGUSTUS (14), invoked by BUSCO, were performed using training data from Cladosporium sphaerospermum (15).

### Data availability.

This whole-genome shotgun project has been deposited in DDBJ/ENA/GenBank under the accession no. JAAQHG000000000. Raw data were deposited in SRA under accession no. SRR11273615 (Bioproject PRJNA610946). The version described in the manuscript is the first version, JAAQHG010000000.
